# Dietary patterns derived by reduced rank regression, macronutrients as response variables, and variation by economic status: NHANES 1999–2018

**DOI:** 10.1007/s00394-024-03501-z

**Published:** 2024-09-17

**Authors:** Samuel C. Coxall, Frances EM. Albers, Sherly X. Li, Zumin Shi, Allison M. Hodge, Brigid M. Lynch, Yohannes Adama Melaku

**Affiliations:** 1 Cancer Epidemiology Division, Cancer Council Victoria, Level 8, 200 Victoria Parade, East Melbourne, Melbourne, VIC 3002 Australia; 2 Centre for Epidemiology and Biostatistics, Melbourne School of Population and Global Health, University of Melbourne, Melbourne, Australia; 3 Medical Research Council Epidemiology Unit, University of Cambridge, Cambridge, UK; 4 Human Nutrition Department, College of Health Sciences, QU Health, Qatar University, Doha, Qatar; 5 Physical Activity Laboratory, Baker Heart and Diabetes Institute, Melbourne, Australia; 6 FHMRI Sleep Health (Adelaide Institute for Sleep Health), College of Medicine and Public Health, Flinders University, Bedford Park, Adelaide, South Australia Australia

**Keywords:** Dietary patterns, Reduced rank regression, Macronutrients, Economic status, Inflammation, Obesity

## Abstract

**Purpose:**

Macronutrient intakes vary across people and economic status, leading to a disparity in diet-related metabolic diseases. This study aimed to provide insight into this by: (1) identifying dietary patterns in adults using reduced rank regression (RRR), with macronutrients as response variables, and (2) investigating the associations between economic status and macronutrient based dietary patterns, and between dietary patterns with central obesity (waist circumference) and systemic inflammation (C-reactive protein [CRP]).

**Methods:**

41,849 US participants from the National Health and Nutrition Examination Survey (NHANES), 1999–2018 were included. The percentages of energy from protein, carbohydrates, saturated fats, and unsaturated fats were used as response variables in RRR. Multivariable generalized linear models with Gaussian distribution were employed to investigate the associations.

**Results:**

Four dietary patterns were identified. Economic status was positively associated with both the high fat, low carbohydrate [β_HighVsLow_ = 0.22; 95% CI: 0.16, 0.28] and high protein patterns [β_HighVsLow_ = 0.07; 95% CI: 0.03, 0.11], and negatively associated with both the high saturated fat [β_HighVsLow_ = -0.06; 95% CI: -0.08, -0.03] and the low alcohol patterns [β_HighVsLow_ = -0.08; 95% CI; -0.10, -0.06]. The high saturated fat pattern was positively associated with waist circumference [β_Q5VsQ1_ = 1.71; 95% CI: 0.97, 2.44] and CRP [β_Q5VsQ1_ = 0.37; 95% CI: 0.26, 0.47].

**Conclusion:**

Macronutrient dietary patterns, which varied by economic status and were associated with metabolic health markers, may explain associations between economic status and health.

**Supplementary Information:**

The online version contains supplementary material available at 10.1007/s00394-024-03501-z.

## Introduction

**Fig. 1:**
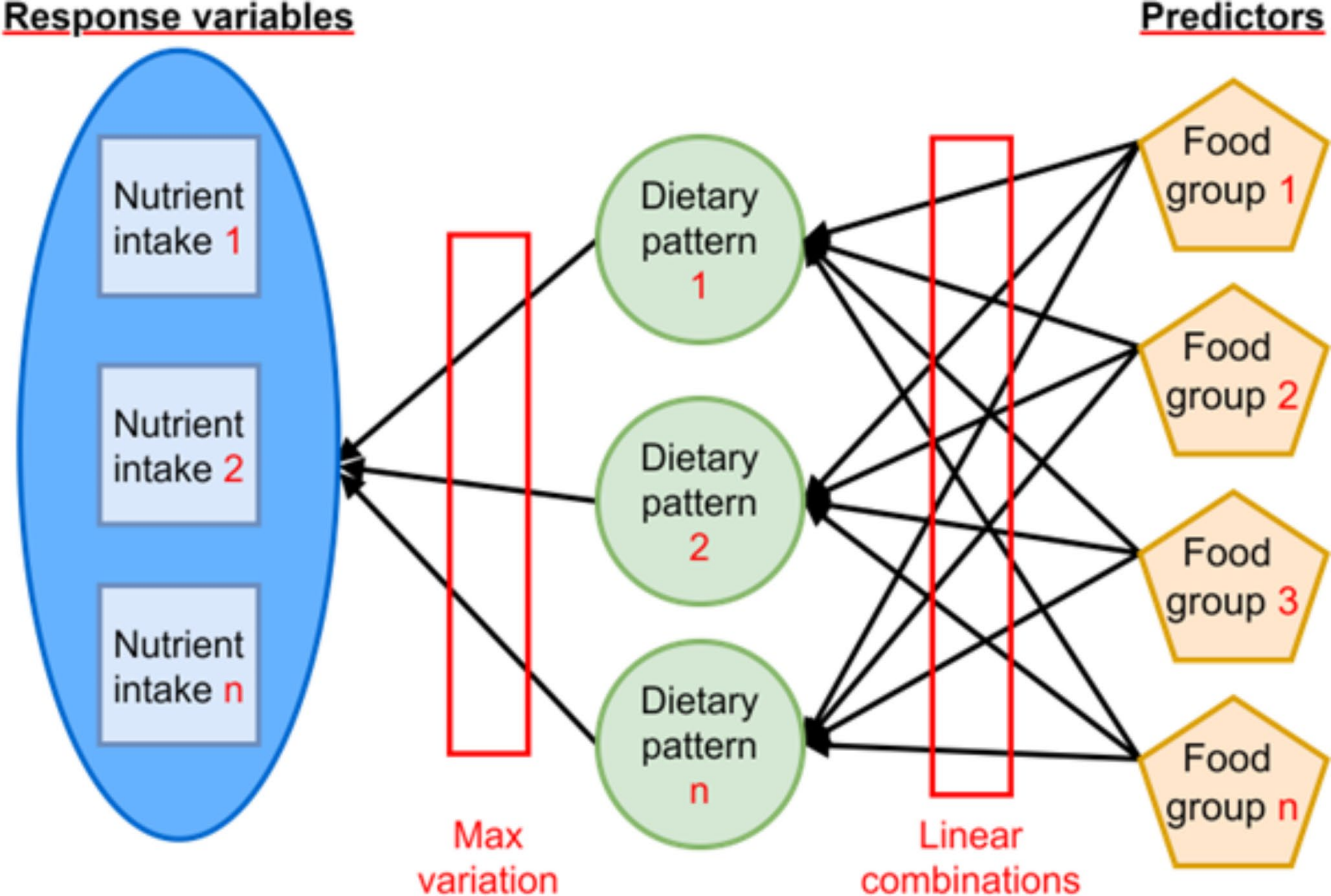
The reduced rank regression model Note: Food groups and nutrient intakes were selected as examples for predictors and response variables, respectively

Dietary habits have been identified as a primary risk factor for morbidity and mortality (1). Thus, the analysis of dietary data is pivotal for designing interventions for the prevention of non-communicable diseases. Traditionally, dietary data analyses in epidemiological studies focus on the relationship between diet and disease by examining the effects of single nutrients or foods [1–3]. However, this analysis technique has several limitations, and a more advanced method is required to examine the joint effects and interactions of all nutrients and food components [1]. A promising solution is dietary pattern analysis, which has three major approaches: a priori,* a posteriori*, and a hybrid of these two methods [2, 4]. Specifically, a hybrid approach utilizes both prior knowledge to define appropriate response variables and observed data to extract dietary patterns [2]. Reduced rank regression (RRR) is the most commonly used hybrid approach in the field of dietary pattern analysis [4]. This statistical method identifies linear combinations of predictors (such as food groups) that maximally explain variation in intermediate response variables (such as biomarkers or nutrient intakes) (Fig. 1) [3]. With adequate prior knowledge, the dietary patterns identified through RRR are based on response variables which are believed to be on the causal pathway between food intakes and the outcome of interest [5, 6].

Evidence shows that macronutrient intakes differ according to economic status, with a common example being a higher consumption of carbohydrates in people with a lower income [7, 8]. Furthermore, numerous studies have found that people of a lower economic status are more likely to follow dietary patterns that are considered unhealthy, such as a “processed foods” pattern characterized by high levels of refined carbohydrates and saturated fat [9, 10]. This may explain why people of lower economic position are likely to have increased risks of metabolic diseases like obesity [11] and systemic inflammation [12], which may lead to long-term health problems such as cardiovascular disease and cancer [13].

Few studies have explored the association between economic status and dietary patterns derived from RRR. One study in pregnant Chinese women [14] used both macro- and micro-nutrients as response variables and found that socio-demographically disadvantaged pregnant women had a lower adherence to a dietary pattern high in protein and haem iron and low in carbohydrate and the ratio of unsaturated fat: saturated fat relative to better off women. Macronutrient intakes may be an important driver of the disparity in diet-related diseases across economic position strata, but few studies have explored these relationships using RRR. An RRR-derived dietary pattern that was positively correlated with saturated fat intake was associated with a higher risk of abdominal obesity in one study [6], but the association was less clear in another study [15]. RRR has also been utilized to explore the association between diet and chronic inflammation [16], however no study has focused on macronutrient intakes [17]. RRR could provide valuable insight into how dietary patterns with different amounts of macronutrients vary across people within different economic statuses, and how this relates to metabolic markers of disease.

The primary aims of this study were to determine population-level dietary patterns for adults from the United States (US) using RRR with macronutrient intakes as response variables, and to investigate the association between economic status and the identified dietary patterns. The secondary aim of this study was to examine the associations of the dietary pattern scores with central obesity and systemic inflammation as markers of health or risk of future illness.

## Methods

### Study design and population

**Fig. 2:**
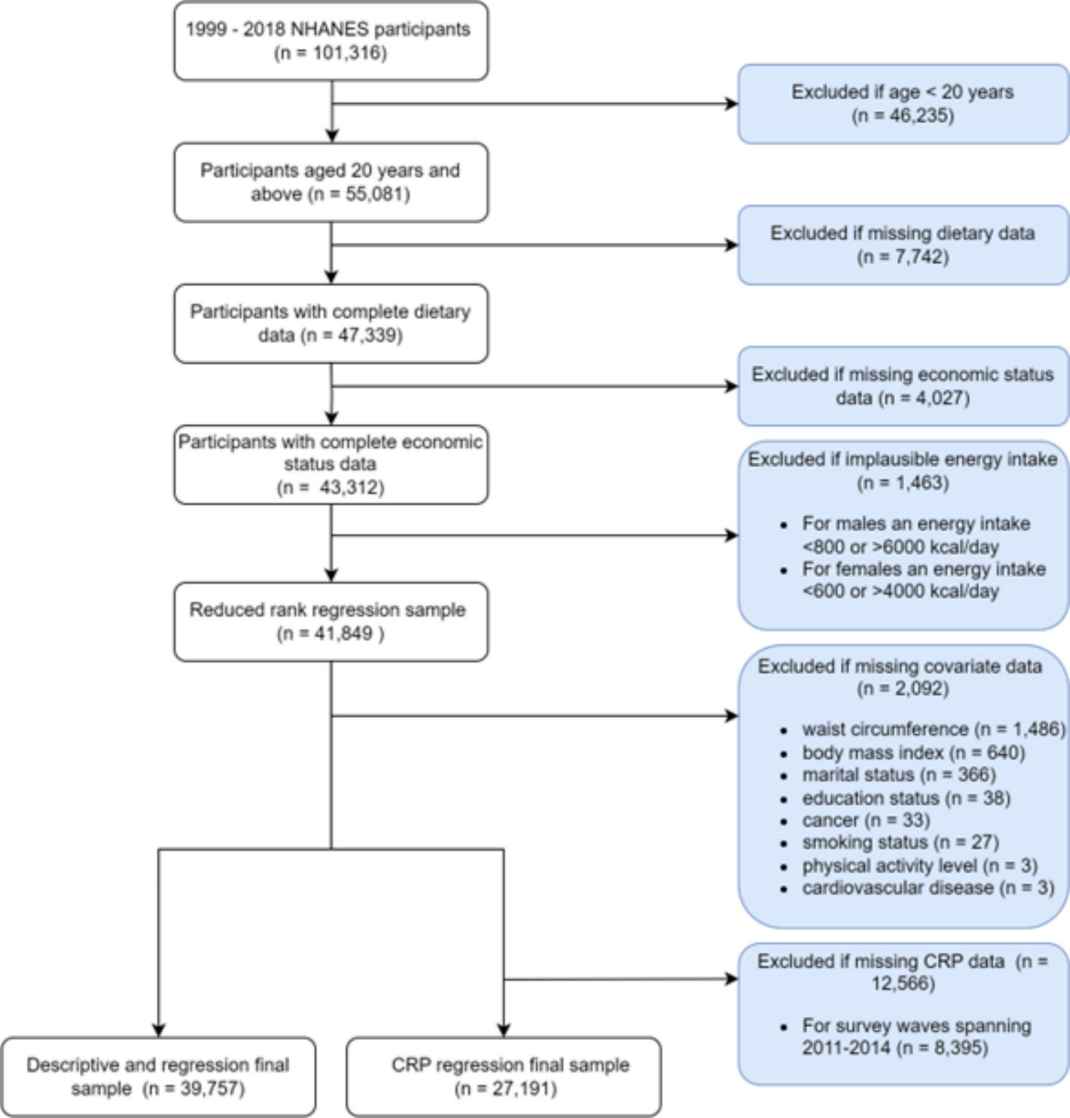
Sampling description of study participants, the National Health and Nutrition Examination Survey (NHANES) Note: CRP, C-reactive protein

We analysed data from the National Health and Nutrition Examination Survey (NHANES), which is an ongoing national repeated cross-sectional survey designed to assess the health and nutritional status of the US population [18]. NHANES is conducted by the National Center for Health Statistics of the Centers for Disease Control and Prevention [19]. In this study, we used data from 2-year NHANES survey cycles conducted during 1999–2018. The included participants were 20 years of age and older (*n* = 55,081). A total of 41,849 participants (49.8% males) were included in the RRR model after exclusions for missing dietary and economic status data, as well as implausible energy intakes (for males an energy intake < 800 or > 6000 kcal/day; for females an energy intake < 600 or > 4000 kcal/day [20]. In the descriptive and regression analyses, there was a total of 39,757 (49.0% males) participants following the exclusion of participants with missing covariate data. An additional 12,566 participants were excluded from the C-reactive protein (CRP) regression analysis as CRP was not measured during the 2011–2014 NHANES survey cycles (Fig. 2). All participants signed written informed consent with approval from the National Center for Health Statistics Research Ethics Review Board. Additional low risk ethical approval was obtained from the Flinders University Human Research Ethics Committee (6547).

### Dietary data

The 1999–2018 NHANES cycles collected dietary data through 24-hour dietary recall interviews using the Automated Multiple-Pass Method [21]. The Automated Multiple-Pass Method is a computer-assisted interview system developed by the United States Department of Agriculture (USDA) for the estimation of food intakes [22]. For the 2003–2018 waves, there were two 24-hour dietary recall interviews. The first dietary interview was administered in person by a trained interviewer in the Mobile Examination Center, and the second was conducted three to ten days later by telephone. We used the data from the first dietary interview in the primary analysis. Respondents were provided with measuring guides for assistance in estimating the portion sizes of consumed foods and beverages. An updated version of the USDA Food and Nutrient Database for Dietary Studies was used to determine the nutrient values of food items for each 2-year survey period [21]. The USDA Food Patterns Equivalents Database was employed to disaggregate the reported food and beverages into 37 USDA Food Patterns components [21]. Further detail on the dietary data collection method is reported elsewhere [21].

### Dietary patterns

**Fig. 3:**
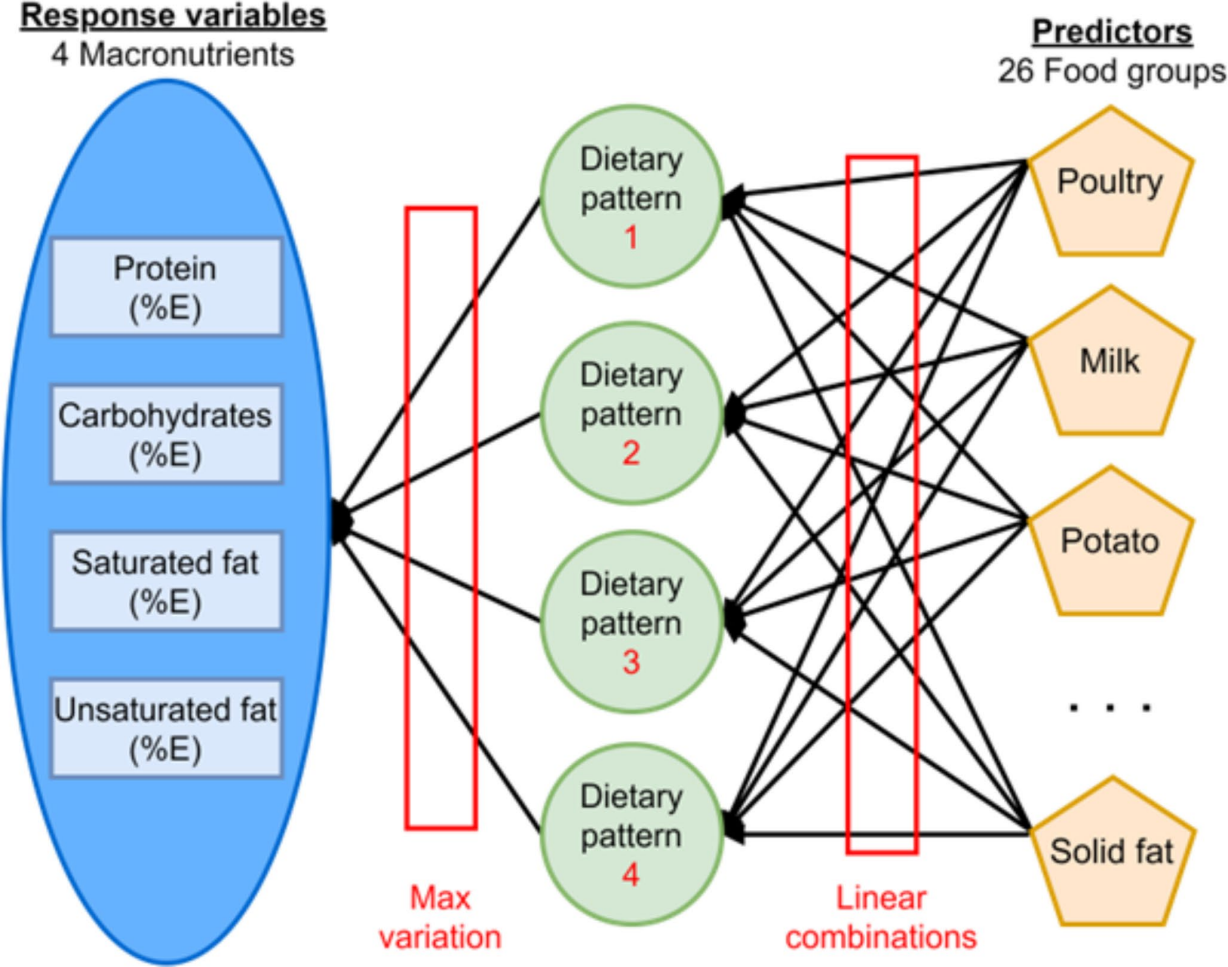
The reduced rank regression model with the response variables and predictors used in this study Note: Only a few of the food groups used in this study are displayed in the figure. %E, percentage of total energy

RRR was used to identify dietary patterns using 26 foods and food groups: citrus fruit; other fruits; dark green vegetables; tomato; potato; other starchy vegetable; other vegetables; whole grain; refined grain; meat, pork and beef; frank meat; organ meat; poultry; fish high in omega-3; fish low in omega-3; egg; soy; nuts; legumes; milk; yogurt; cheese; liquid fat; solid fat; added sugar; and alcohol. The food groupings were based on nutrient composition and cooking methods. Percentages of energy from four macronutrients (protein, carbohydrates, saturated fat, and unsaturated fat) were calculated and used as response variables. The RRR model used in this study is depicted in Fig. 3. The number of dietary patterns derived using RRR is dependent on the number of response variables. Hence, four dietary patterns were extracted in our analysis.

### Economic status

Family poverty to income ratio was the variable selected to operationalize economic status; it is the ratio of family income to the specific poverty threshold for that survey year. These data were obtained through a questionnaire conducted by trained interviewers in the home of NHANES participants using Computer-Assisted Personal Interview methodology [23]. Family poverty to income ratio was classified into three groups based on a published study and the Patient Protection and Affordable Care Act: low (≤ 1), middle (1–4), and high (≥ 4) [24]. These groups were utilized to represent low, medium, and high levels of economic status, respectively.

### Central obesity and systemic inflammation

Central obesity was assessed as waist circumference, which is a measure of abdominal adiposity independently associated with an increased risk of morbidity and mortality [25]. Waist circumference was measured with a tape measure at the uppermost lateral border of the hip crest (ilium) to the nearest 0.1 cm [26, 27]. These measures were undertaken by trained health technicians in the Mobile Examination Center during the examination segment of the NHANES survey cycles. Waist circumference was analysed as a continuous variable.

Blood samples were obtained from participants as a component of NHANES. CRP, a biomarker of systemic inflammation, was measured in the 1999–2010, 2015–2016 and 2017–2018 survey cycles, but not the 2011–2014 survey cycle. The 1999–2010 survey cycles used latex-enhanced nephelometry to measure CRP in mg/dL. This method is based on the reaction between a soluble analyte and its corresponding antigen or antibody bound to polystyrene particles. Quantification of CRP occurs through anti-CRP antibodies covalently linking with the polystyrene core and hydrophilic shell of CRP particles [23]. Two alternative methodologies of a higher sensitivity were used to measure CRP levels in the 2015–2016 survey cycle and 2017–2018 survey cycle: a near infrared particle immunoassay rate method and a two-reagent immunoturbidimetric system, respectively. CRP was modelled as a continuous variable in mg/L, and levels above 10.0 mg/L were excluded because this may indicate acute infections [28].

### Confounders

Sociodemographic factors, behavioural factors and chronic conditions were included as confounding variables. Confounders were selected based on directed acyclic graphs (DAGs) of the relationships between economic status and dietary pattern score, dietary pattern score and abdominal obesity, and dietary pattern score and systemic inflammation (Supplementary Figs. 1, 2 and 3).

The sociodemographic characteristics were: age (years), sex (male or female), ethnicity (Mexican American, other Hispanic, non-Hispanic white, non-Hispanic black, or other races including multi-racial), marital status (married/living with partner, widowed, divorced/separated, or never married), and education level (less than high school, high school diploma or equivalent, or more than high school). The behavioural factors included smoking status, physical activity, and total energy intake. Smoking status was categorized as: never, former (does not currently smoke but has smoked > 100 cigarettes in lifetime), or current (currently smokes and has smoked > 100 cigarettes in lifetime). Physical activity was evaluated using metabolic equivalent of task (MET)-minutes, which was calculated through multiplying the weekly minutes for each moderate to vigorous activity by its appropriate MET score. Participants were categorized into three groups of physical activity level: low (< 600 MET-minutes per week), moderate (600 to < 1200 MET-minutes per week), or high (≥ 1200 MET-minutes per week). Total energy intake from foods and beverages was computed in kcal/day and then converted and reported in kJ/day using the following equation: 1 kcal = 4.184 kJ.

Diabetes (yes or no), cardiovascular disease (yes or no), and cancer (yes or no) were also included as confounding variables. Diabetes was defined as meeting at least one of the following criteria: a fasting plasma glucose ≥ 126 mg/dL; a random plasma glucose ≥ 200 mg/dL with symptoms and signs present (e.g., diabetes retinopathy); a 2-hour plasma glucose ≥ 200 mg/dL during a 75 g oral glucose tolerance test; and/or a haemoglobin A1c level ≥ 6.5%. Participants could also be classified as having diabetes if they gave a positive response to any of the following questions: “Did a doctor tell you, you have diabetes?”, “Are you taking insulin?”, and/or “Do you take pills to lower blood sugar?” [29]. Participants were also asked to self-report whether they had been diagnosed with cardiovascular disease or cancer by a doctor (yes or no). Models for economic status and dietary patterns were adjusted for sex, age, marital status, ethnicity, educational status, smoking status, physical activity level, total energy intake, diabetes, cardiovascular disease, and cancer. For the association of dietary patterns with waist circumference, models were additionally adjusted for economic status. For the association of dietary patterns with CRP, models were additionally adjusted for economic status and the method of CRP measurement (survey cycles).

### Statistical analysis

All analyses accounted for the complex survey design using NHANES-assigned dietary data weights, population sampling units, and strata. Data were downloaded from the Centers for Disease Control and Prevention website [30]. Descriptive analysis of sociodemographic and lifestyle characteristics was performed across quintiles of dietary pattern scores. Characteristics were summarized using mean (standard deviation [SD]) for symmetrically distributed continuous variables, median (interquartile range [IQR]) for skewedly distributed continuous variables, and proportion for categorical variables.

Four dietary patterns were identified using RRR. Factor loadings, which are the standardized correlations between food groups and the dietary patterns (factors), were calculated. The proportions of factor-specific and all factor variances that explain the response variables and food groups were determined. Participants received a factor score for each dietary pattern which represents their adherence to the dietary pattern. This was derived in the form of a continuous variable that evaluates how much of a participant’s diet approximates the corresponding dietary pattern [2]. Factor scores were divided into quintiles (Q1 [lowest intake], Q2, Q3, Q4 and Q5 [highest intake]) for further analyses. Correlations (response scores) between the response variables and dietary patterns were quantified.

The cross-sectional associations between economic status and dietary pattern scores were determined using generalized linear models with Gaussian distribution and identity link. The models were adjusted for potential confounders described above. The associations of dietary pattern scores with central adiposity (represented by waist circumference) and systemic inflammation (represented by CRP) were determined using multivariable generalized linear models with Gaussian distribution and identity link. A supplementary analysis with stratification by sex was performed on the association between dietary pattern scores and central adiposity. The trend of association across quintiles of each dietary pattern were assessed using quintiles as a continuous parameter. All statistical analyses were performed using Stata statistical software version 17.0 (Statacorp, College Station, TX). A Stata module was installed to perform RRR [31].

## Results

### Characteristics of study participants

The characteristics of the study participants are shown in Table 1. The median age of the participants was 46 years (IQR: 33.0, 59.0). More than two-thirds (69.9%) of the participants were non-Hispanic white. Most study participants were married or living with a partner (63.4%) and had an educational status greater than high school (60.1%). Over half of the study participants were non-smokers at the time of data collection (53.4%) and over half were meeting recommended physical activity levels (51.4%). The prevalence of diabetes, cardiovascular disease, and cancer were 12.2%, 8.5%, and 9.6%, respectively. Overall, 14.0% of participants were classified as low economic status, whereas 48.9% and 37.1% were considered as medium and high economic status, respectively. The study participants had a mean waist circumference of 98.4 cm (SD: 16.3). The median CRP level in the 1999–2010, 2015–2016, and 2017–2018 survey cycles was 1.6 mg/L (IQR: 0.7, 3.3), 1.6 mg/L (IQR: 0.6, 3.4), and 1.7 mg/L (IQR: 0.8, 3.4), respectively (Table 1).

**Table 1:** Characteristics of study participants across the first and last quintile of each dietary pattern among adults attending NHANES 1999–2018 (*n* = 39,757)

	Overall	High fat, low carbohydrate (first) pattern		High protein (second) pattern		High saturated fat (third) pattern		Low alcohol (fourth) pattern	
		Q1 (*n* = 7,952)	Q5 (*n* = 7,951)	Q1 (*n* = 7,952)	Q5 (*n* = 7,951)	Q1 (*n* = 7,952)	Q5 (*n* = 7,951)	Q1 (*n* = 7,952)	Q5 (*n* = 7,951)
Age (years), median (IQR)	46.0 (33.0, 59.0)	43.0 (31.0, 56.0)	46.0 (33.0, 58.0)	45.0 (32.0, 57.0)	45.0 (33.0, 58.0)	47.0 (34.0, 58.0)	42.0 (31.0, 56.0)	44.0 (33.0, 56.0)	48.0 (35.0, 59.0)
Sex, %									
Male	49.0	53.8	60.1	53.6	58.3	57.2	57.0	62.3	55.9
Female	51.0	46.2	39.9	46.4	41.7	42.8	43.0	37.7	44.1
Marital status, %									
Married or living with a partner	63.4	61.3	66.0	62.6	64.5	65.1	61.4	63.1	66.9
Separated/divorced	13.0	13.1	12.4	14.1	12.1	11.8	14.0	14.3	11.1
Widowed	5.6	4.6	4.0	4.9	4.4	4.3	4.4	3.5	4.7
Never married	18.0	21.1	17.6	18.4	19.0	18.8	20.2	19.1	17.2
Educational status, %									
Less than high school	15.8	20.1	12.5	14.0	16.4	12.2	16.4	12.0	13.3
High school diploma (including GED)	24.1	24.9	22.8	27.5	20.7	20.7	27.0	22.9	20.8
More than high school	60.1	55.0	64.7	58.5	62.9	67.1	56.6	65.1	65.9
Ethnicity, %									
Mexican American	7.9	11.2	6.5	6.1	9.7	7.8	7.4	5.9	9.0
Non-Hispanic White	69.9	65.1	74.4	74.7	64.5	65.8	76.8	76.4	65.9
Non-Hispanic Black	10.5	9.7	10.3	11.3	10.0	11.8	8.3	9.3	9.3
Other Hispanic	5.1	6.1	3.8	3.5	6.9	5.0	4.1	4.1	5.4
Other race – including Multi-Racial	6.6	8.0	5.1	4.5	9.0	9.6	3.5	4.3	10.4
Economic status, %									
Low	14.0	17.7	10.8	13.7	13.5	11.9	14.4	10.4	12.6
Medium	48.9	50.3	45.5	50.0	46.2	45.3	52.3	43.3	46.6
High	37.1	32.1	43.7	36.2	40.3	42.8	33.3	46.3	40.8
Smoking status, %									
Non-smoker	53.4	53.5	50.4	49.3	56.3	53.2	50.4	41.1	59.8
Ex-smoker	25.1	21.1	28.2	25.4	26.3	27.8	23.3	28.5	26.7
Current smoker	21.5	25.3	21.4	25.4	17.4	19.0	26.4	30.4	13.4
Physical activity level, %									
Low	36.0	34.3	32.9	35.4	32.5	31.0	36.0	29.7	31.1
Moderate	12.6	12.8	12.4	12.2	12.5	11.3	12.4	13.1	11.5
High	51.4	52.9	54.7	52.4	55.0	57.7	51.7	57.3	57.3
Diabetes, %									
Yes	12.2	9.0	13.6	10.0	12.3	11.3	10.7	7.8	12.4
No	87.8	91.0	86.4	90.1	87.7	88.7	89.3	92.2	87.6
Cardiovascular disease, %									
Yes	8.5	8.1	7.6	7.8	7.7	6.8	7.6	6.1	7.9
No	91.5	91.9	92.4	92.3	92.4	93.2	92.4	93.9	92.1
Cancer, %									
Yes	9.6	7.5	8.7	9.5	8.6	8.5	8.6	8.6	9.5
No	90.4	92.5	91.3	90.5	91.4	91.6	91.4	91.4	90.5
BMI (kg/m^2^), mean (SD)	28.7 (6.6)	28.2 (6.8)	29.6 (6.5)	28.8 (6.6)	28.7 (6.6)	28.4 (6.4)	29.0 (6.6)	27.7 (5.6)	28.5 (6.4)
Total energy intake (kJ/day), mean (SD)	9045.5 (3732.4)	9487.0 (3944.8)	10905.2 (3656.6)	11482.7 (3723.5)	9024.3 (3759.4)	10244.5 (3770.9)	10603.6 (3783.8)	10290.7 (3804.2)	10495.7 (3756.8)
Percent of energy from protein, mean (SD)	15.7 (5.1)	12.8 (4.2)	17.8 (5.2)	11.3 (2.5)	22.3 (5.2)	15.7 (5.5)	15.6 (4.2)	14.6 (4.3)	16.8 (5.6)
Protein (gm/day), mean (SD)	83.1 (40.1)	71.9 (36.6)	112.9 (43.0)	78.8 (33.4)	116.3 (47.2)	93.7 (42.9)	97.5 (41.0)	88.5 (39.7)	103.0 (44.5)
Percent of energy from carbohydrates, mean (SD)	48.4 (11.4)	62.0 (9.1)	36.2 (7.0)	48.9 (10.2)	44.7 (11.7)	44.3 (10.7)	47.5 (10.2)	41.0 (9.6)	51.1 (11.2)
Carbohydrates (gm/day), mean (SD)	259.0 (118.1)	344.2 (136.4)	241.1 (103.7)	334.5 (128.0)	239.6 (114.2)	271.0 (118.9)	301.1 (125.9)	252.1 (110.6)	318.6 (131.3)
Percent of energy from unsaturated fat, mean (SD)	19.9 (6.3)	13.7 (4.2)	25.8 (5.6)	23.4 (6.3)	17.4 (5.5)	24.2 (7.3)	18.6 (4.6)	18.2 (5.6)	21.7 (7.3)
Unsaturated fat (gm/day), mean (SD)	48.4 (26.4)	35.6 (20.3)	73.9 (27.4)	70.5 (28.2)	42.3 (23.3)	65.0 (30.2)	52.9 (24.2)	50.3 (25.4)	61.0 (30.9)
Percent of energy from saturated fat, mean (SD)	14.2 (4.6)	9.9 (3.3)	18.4 (4.0)	15.6 (4.4)	13.1 (4.5)	12.2 (3.6)	17.7 (4.3)	13.6 (4.7)	12.5 (3.6)
Saturated fat (gm/day), mean (SD)	34.6 (19.1)	25.7 (15.1)	52.6 (19.9)	47.5 (20.1)	32.0 (18.5)	33.4 (16.7)	49.4 (20.6)	37.7 (20.0)	35.7 (18.6)
Waist circumference (cm), mean (SD)	98.4 (16.3)	96.9 (16.7)	101.3 (16.0)	99.1 (16.2)	98.3 (16.5)	98.0 (15.9)	99.7 (16.2)	97.2 (14.5)	98.0 (16.4)
CRP in the 1999–2010 survey cycles (mg/L), median (IQR)^**a**^	1.6 (0.7, 3.3)	1.4 (0.6, 3.1)	1.6 (0.7, 3.4)	1.6 (0.7, 3.3)	1.5 (0.6, 3.2)	1.2 (0.5, 2.8)	1.7 (0.7, 3.5)	1.3 (0.6, 2.8)	1.3 (0.6, 2.9)
CRP in the 2015–2016 survey cycle (mg/L), median (IQR)^**a**^	1.6 (0.6, 3.4)	1.6 (0.6, 3.5)	1.5 (0.6, 3.0)	1.7 (0.7, 3.7)	1.4 (0.6, 2.9)	1.4 (0.6, 3.0)	1.8 (0.8, 3.7)	1.4 (0.6, 2.9)	1.3 (0.6, 3.0)
CRP in the 2016–2017 survey cycle (mg/L), median (IQR)^**a**^	1.7 (0.8, 3.4)	1.6 (0.9, 3.5)	1.5 (0.8, 3.0)	1.7 (0.8, 3.6)	1.4 (0.8, 2.9)	1.5 (0.7, 3.1)	2.0 (0.9, 3.9)	1.5 (0.8, 3.5)	1.3 (0.7, 3.0)

Data are presented as mean (SD) for normally distributed continuous measures, median (IQR) for non-normally distributed continuous measures, and % for categorical measures

^**a**^ CRP levels are displayed as separate for specific survey cycles as alternative methods for CRP measurement were used

IQR, interquartile range; GED, General Educational Development; BMI, body mass index; SD, standard deviation; CRP, C-reactive protein

### Dietary patterns

We identified four dietary patterns using RRR. Each dietary pattern was named according to their correlation with macronutrient intakes. A dietary pattern was classified to be high or low in a macronutrient intake if the correlation was greater than 0.5 or less than − 0.5, respectively.

**Fig. 4:**
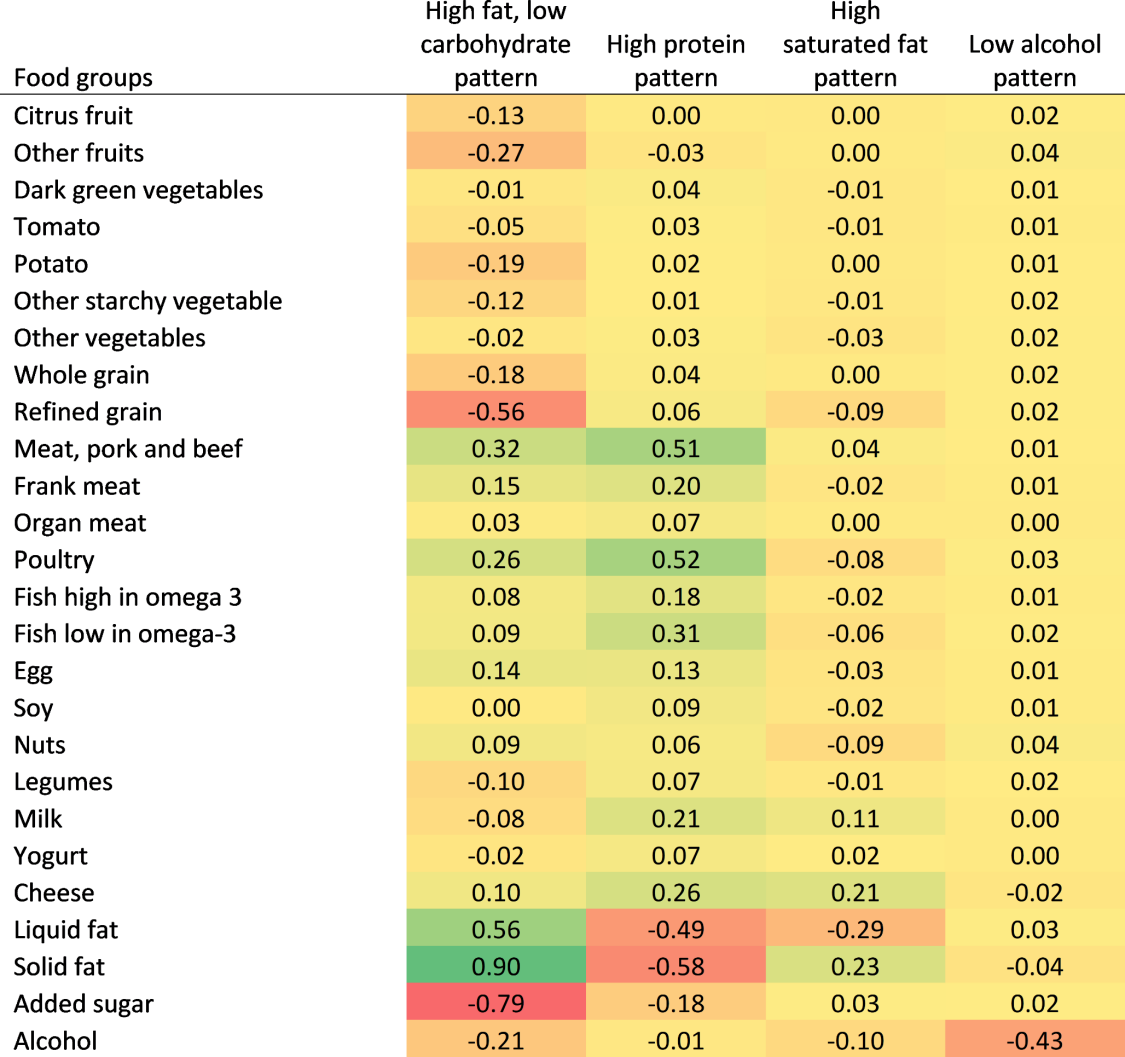
Factor loadings of food groups in each dietary pattern identified using reduced rank regression (*n* = 41,849) Note: The colour gradation indicates the direction and strength of the correlation between the food groups with the corresponding dietary patterns. The colour yellow indicates no correlation. A relatively higher intake of a food group within a dietary pattern is denoted by a deep green colour. A relatively lower intake of a food group within a dietary pattern is denoted by a deep red colour

Figure 4 displays the generated dietary patterns and the factor loadings of food groups. The first pattern was named the high fat, low carbohydrate pattern and was characterized by high intakes of solid fat, liquid fat, meat (pork and beef), and poultry, as well as low intakes of added sugar, refined grain, fruits, and vegetables. The second pattern, termed high protein pattern, was characterized by high consumption of meat (pork and beef), poultry, fish, egg, milk, and cheese, together with low intake of liquid and solid fat. The third pattern (high saturated fat pattern) was primarily characterized by high intakes of solid fat, cheese, and milk, as well as low consumption of liquid fat. The fourth pattern was predominately distinguished by a low consumption of alcohol and moderate consumption of macronutrients. We named this pattern the low alcohol pattern.

Table 2 depicts the explained variation in food intakes and response variables for each of the identified dietary patterns. The four factors explained 72.2% of the response variable variation (percentage of energy from protein, carbohydrates, saturated fats, and unsaturated fats). The first two factors – the high fat, low carbohydrate pattern and high protein pattern – explained 40.7% and 19.3% of the variability in the response variable intakes, respectively. Percentage of energy from carbohydrates was the most explained response in the high fat, low carbohydrate pattern (62.6%). In the high protein pattern, the largest explained variation (59.8%) was observed in the percentage of energy from protein. We found 16.3% of the variation in predictors (food groups) was explained by the factors.

**Table 2:** Explained variation (%) in food intakes and response variables for each dietary pattern identified using reduced rank regression (*n* = 41,849)

Dietary pattern	Response variables, %E					Food intakes
	Protein	Carbohydrates	Saturated fat	Unsaturated fat	Total	
High fat, low carbohydrate pattern	11.1	62.6	42.3	46.7	40.7	3.5
High protein pattern	59.8	1.8	4.2	11.4	19.3	3.1
High saturated fat pattern	0.0	0.8	18.4	9.2	7.1	5.5
Low alcohol pattern	3.2	11.5	0.8	4.9	5.1	4.2
Total	74.1	76.7	65.7	72.2	72.2	16.3

%E, percentage of total energy

**Fig. 5:**
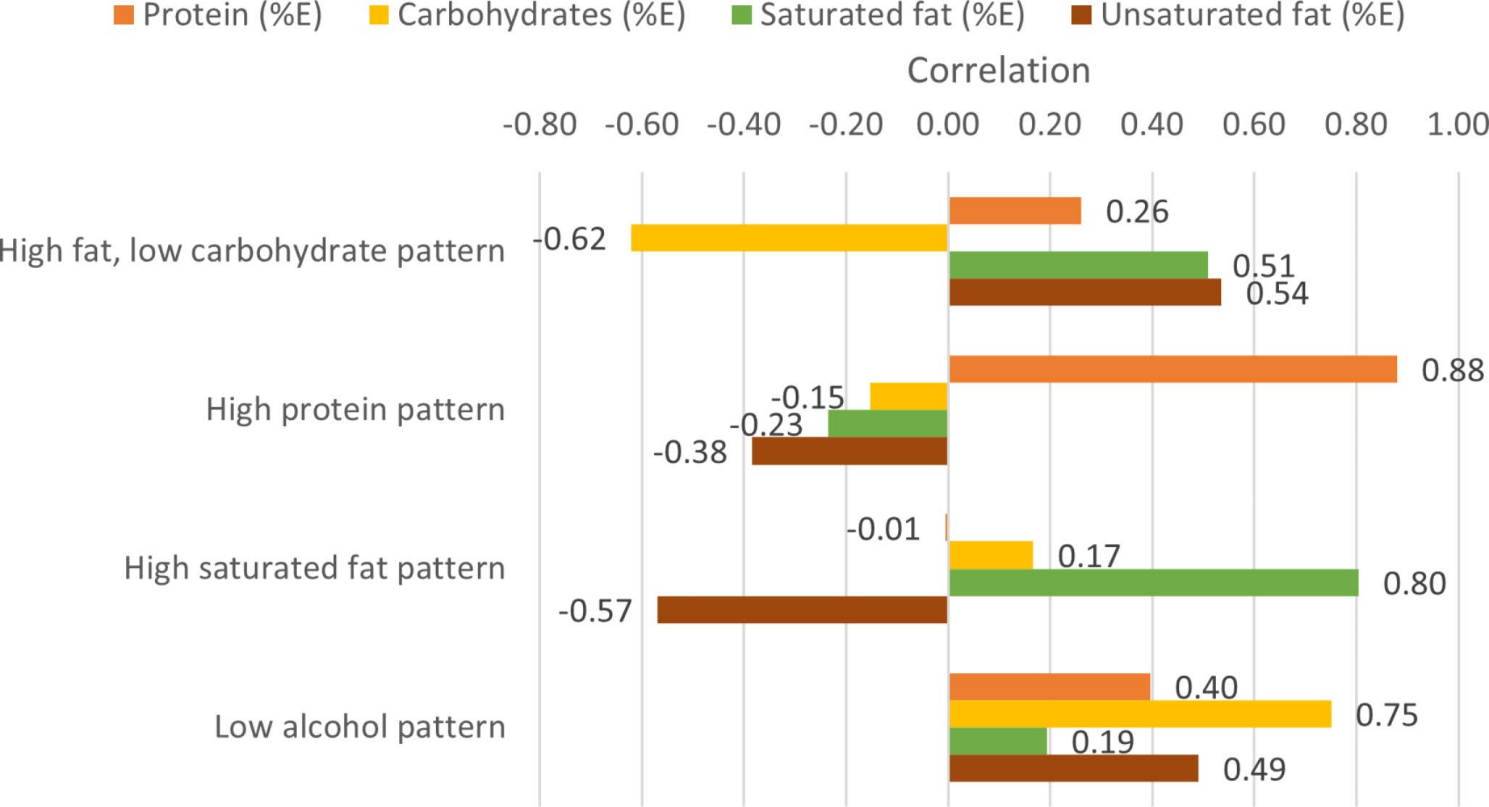
Correlation (response scores) between factors and response variables obtained from reduced rank regression (*n* = 41,849) Note: %E, percentage of total energy

The correlation (response scores) between factors and response variables are presented in Fig. 5. The high fat, low carbohydrate pattern (factor 1) was positively correlated with energy from unsaturated fat, saturated fat, and protein (0.54, 0.51 and 0.26, respectively). Factor 1 also had a significant negative correlation (-0.62) with carbohydrate energy. There was a substantial positive correlation (0.88) between protein energy and the high protein pattern. The percentage of energy from saturated fat was positively correlated (0.80) with the high saturated fat pattern; in contrast, unsaturated fat energy was negatively correlated (-0.57). The low alcohol pattern was positively correlated with all the response variables (Fig. 5).

### Characteristics of study participants across dietary pattern quintiles

Table 1 displays the characteristics of the study participants across the first and last quintile of the four dietary patterns. We observed greater variability in economic status across quintiles of the high fat, low carbohydrate pattern and high saturated fat pattern. The same trend was observed for educational status. A larger proportion of participants were male in the first and last quintiles of all the dietary patterns. There were considerable differences in the ethnicity of the participants across the quintiles for each dietary pattern. The fifth quintile of the low alcohol pattern, compared to the first quintile, had a notably lower percentage of current smokers (13.4% vs. 30.4%) and higher percentage of non-smokers (59.8% vs. 41.1%). The characteristics of the study participants across all the quintiles for each dietary pattern are provided in Supplementary Tables 1, 2, 3 and 4.

### Dietary patterns and economic status

Table 3 presents the associations of economic status with dietary pattern scores, after adjusting for confounders. For the high fat, low carbohydrate pattern (factor 1), the dietary pattern scores increased with rising level of economic status; participants with medium and high economic status had 0.09 (95% CI: 0.04, 0.15) and 0.22 (95% CI: 0.16, 0.28) higher factor scores than those with low economic status, respectively. In contrast, factor scores decreased with increasing level of economic status for the low alcohol pattern (factor 4); the β-coefficients for medium and high economic status were − 0.03 (95% CI; -0.05, -0.01) and − 0.08 (95% CI; -0.10, -0.06), respectively. For the high saturated fat pattern (factor 3), those with high economic status were observed to have a lower dietary pattern score compared to those with low economic status (β-coefficient for high economic status: -0.06; (95% CI: -0.08, -0.03). Participants with high economic status had a 0.07 (95% CI: 0.03, 0.11) larger factor score for the high protein pattern (factor 2) than those with low economic status (Table 3).

**Table 3:** Coefficients () and 95% confidence intervals of dietary pattern scores across levels of economic status, after adjusting for confounders (*n* = 39,757)

Dietary patterns	β (95% CI)			*P* for trend
	Low economic status	Medium economic status	High economic status	
High fat, low carbohydrate pattern	Ref.	0.09 (0.04, 0.15)	0.22 (0.16, 0.28)	< 0.001
High protein pattern	Ref.	0.01 (-0.02, 0.05)	0.07 (0.03, 0.11)	< 0.001
High saturated fat pattern	Ref.	0.01 (-0.01, 0.04)	-0.06 (-0.08, -0.03)	< 0.001
Low alcohol pattern	Ref.	-0.03 (-0.05, -0.01)	-0.08 (-0.10, -0.06)	< 0.001

Adjusted for sex, age, marital status, ethnicity, educational level, smoking status, physical activity level, total energy intake, diabetes, cardiovascular disease, and cancer

*P for trend* was calculated by including economic status as a continuous variable

### Dietary patterns, systemic inflammation, and central obesity

The associations for quintiles of dietary pattern scores with waist circumference and CRP are provided in Table 4. Waist circumference increased across the quintiles of the high fat, low carbohydrate pattern and the high saturated fat pattern. Participants in Q5 of the high fat, low carbohydrate pattern and the high saturated fat pattern had a 3.16 cm (95% CI: 2.35, 3.98) and a 1.71 cm (95% CI: 0.97, 2.44) higher waist circumference compared to those in Q1, respectively. The associations stratified by sex for quintiles of dietary pattern scores with waist circumference demonstrated similar findings (Supplementary Table 5). CRP increased across the quintiles of the high saturated fat pattern and the high fat, low carbohydrate pattern; those in Q5 of the high saturated fat pattern and the high fat, low carbohydrate pattern had a 0.37 mg/L (95% CI: 0.26, 0.47) and a 0.13 mg/L (95% CI: 0.01, 0.25) higher CRP level than those in Q1, respectively (Table 4).

**Table 4:** Coefficients () and 95% confidence intervals of waist circumference (*n* = 39,757) and C-reactive protein level (*n* = 27,191) across quintiles of dietary pattern scores, after adjusting for confounders

Dietary patterns	β (95% CI)					*P* for trend
	Q1	Q2	Q3	Q4	Q5	
**Waist circumference (cm)** ^**a**^						
High fat, low carbohydrate pattern	Ref.	-0.34 (-1.03, 0.34)	0.59 (-0.06, 1.25)	1.46 (0.76, 2.17)	3.16 (2.35, 3.98)	< 0.001
High protein pattern	Ref.	0.06 (-0.60, 0.72)	-0.59 (-1.22, 0.04)	-0.47 (-1.21, 0.27)	-0.55 (-1.21, 0.12)	0.033
High saturated fat pattern	Ref.	0.17 (-0.58, 0.92)	0.80 (0.02, 1.57)	0.70 (-0.04, 1.43)	1.71 (0.97, 2.44)	< 0.001
Low alcohol pattern	Ref.	2.73 (2.10, 3.36)	2.53 (1.82, 3.23)	1.81 (1.11, 2.50)	0.40 (-0.31, 1.11)	0.835
**C-reactive protein (mg/L)** ^**b**^						
High fat, low carbohydrate pattern	Ref.	-0.02 (-0.12, 0.09)	0.04 (-0.07, 0.15)	0.09 (-0.01, 0.19)	0.13 (0.01, 0.25)	0.006
High protein pattern	Ref.	-0.05 (-0.15, 0.05)	-0.07 (-0.18, 0.04)	-0.12 (-0.23, 0.00)	-0.11 (-0.22, -0.01)	0.016
High saturated fat pattern	Ref.	0.03 (-0.07, 0.13)	0.23 (0.11, 0.34)	0.27 (0.14, 0.40)	0.37 (0.26, 0.47)	< 0.001
Low alcohol pattern	Ref.	0.32 (0.18, 0.46)	0.19 (0.06, 0.33)	0.23 (0.11, 0.34)	-0.08 (-0.19, 0.02)	0.033

^**a**^ Waist circumference models were adjusted for sex, age, educational status, marital status, ethnicity, economic status, smoking status, physical activity level, total energy intake, diabetes, cardiovascular disease, and cancer

^**b**^ C-reactive protein models were additionally adjusted for survey cycles

*P for trend* was calculated by including the quintiles of factor scores as continuous variables

## Discussion

In this cross-sectional study using data from NHANES, we identified four dietary patterns using the RRR method with macronutrients as response variables. The first pattern (high fat, low carbohydrate pattern) was positively correlated with energy intake from saturated fat and unsaturated fat, and negatively correlated with energy from carbohydrate. This was explained by high intakes of fatty foods (e.g., butter and oils), as well as low consumption of grains, fruits, and vegetables. The second pattern (high protein pattern) was positively correlated with energy from protein, which was primarily driven by high meat intake. Most of the variation in the response variables was explained by the first two dietary patterns. The third pattern (high saturated fat pattern) was positively correlated with energy from saturated fat, and negatively correlated with energy from unsaturated fat. This was mainly explained by high consumptions of solid fat and cheese, together with low consumption of liquid fat. The fourth pattern (low alcohol pattern) was positively correlated with energy from all the macronutrients.

We found that economic status was positively associated with both scores for the high fat, low carbohydrate and high protein patterns, as well as negatively associated with scores for both the high saturated fat and low alcohol patterns. The high saturated fat and high fat, low carbohydrate patterns were positively associated with central obesity and systemic inflammation.

The dietary patterns derived in our study largely differ from the findings of the few previous studies that have identified dietary patterns using RRR with macronutrients as response variables [14, 32, 33]. This was expected because these studies were conducted in dissimilar populations: pregnant Chinese women [14], African American women [32], and elderly Germans [33]. However, Hoffman et al. [33] derived a similar first factor to ours in elderly Germans, primarily characterized by high consumption of fat, as well as low intake of carbohydrates. In similarity to our study, the dietary patterns derived in each of these studies explained significantly greater variance in the response variables than food groups, which is consistent with the methodology of RRR.

Our findings illustrate that economic status is associated with dietary patterns that are based on macronutrient intakes. This suggests that the relative contribution of macronutrients to energy intake varies among levels of economic status. We found that people of higher economic status were more likely to have dietary patterns that were negatively correlated with energy from carbohydrates. In a study of pregnant women in China, lower income was associated with greater adherence to a dietary pattern positively correlated with carbohydrates [14]. Further, numerous studies have reported that low economic status is linked with higher consumption of carbohydrates [7, 8, 34, 35]. However, none of these studies were conducted in a US population. Our findings also indicate that higher economic status is associated with dietary patterns with relatively higher unsaturated fat intake. Similarly, a study among adolescents in Southern California communities found that participants from higher income families had significantly greater intakes of polyunsaturated fats [36]. However, most studies explored total fat intake rather than the intake of different types of fats, and observed associations were inconsistent. Our results are also suggestive of a small positive association between economic status and a high protein dietary pattern, which has been substantiated by several studies [34–36]. Overall, however, inconsistent results have been reported in previous literature regarding the observed association between economic status and the macronutrient composition of the diet.

Our secondary analyses suggest that the dietary patterns based on macronutrient intakes are associated with abdominal adiposity and systemic inflammation. Central obesity was positively associated with a high saturated fat dietary pattern. A similar conclusion was drawn from a study conducted in adults living in the United Kingdom [6]. We also discovered a positive association between systemic inflammation and a dietary pattern consisting of a relatively high consumption of saturated fat. Saturated fat intake has been linked to inflammation in numerous studies [37–40].

A potential explanation for the link between economic status and diet may be differing prices for food sources of certain macronutrients. Food sources of carbohydrates tend to be less expensive than foods that are rich in protein [41, 42]; lack of affordability may encourage people of lower economic status to consume diets relatively rich in carbohydrates and relatively poor in protein. The positive association that we observed between economic status and score for dietary patterns with relatively higher unsaturated fat intake may be due to health literacy. Better health literacy is reported in people of a higher economic status [43, 44], which suggests that greater awareness of the potential health benefits of unsaturated fats may increase consumption in these individuals.

Several biological mechanisms have been proposed for the positive association observed between a high saturated fat dietary pattern and central adiposity. There is evidence that saturated fats are directly correlated with fat cell size and number [45]. In addition, a genetic variant within the promoter of the apolipoprotein A-II gene may impact the relation between saturated fats and obesity risk [46]. However, the links between diet and central obesity may have a behavioural explanation, as the consumption of certain dietary patterns may be related to lifestyle behaviours associated with central obesity. A positive association between high saturated fat consumption and systemic inflammation is biologically plausible. Chronic consumption of a dietary pattern characterized by high saturated fat could increase systemic inflammation through the repeated promotion of lipopolysaccharide absorption into the bloodstream [39].

The dietary patterns identified using RRR in this study are important for understanding the current landscape of food intake in the US population. Investigating differences in dietary patterns by economic status may assist in recognizing a target population for future public health awareness campaigns and related interventions. Such strategies may consist of dietary advice regarding the distribution of macronutrient consumption, although this requires further research. The findings of this study will contribute to the current debate on how the relative contribution of macronutrient intake affects metabolic health outcomes such as central obesity and inflammation. This may help to inform policy regarding the promotion of healthy dietary behaviours for the prevention of chronic diseases. Additionally, increasing the affordability of foods that are rich in protein and improving the health literacy of people in low economic status are actions that may reduce the disparities in the relative contribution of macronutrient intakes in the diet by economic status.

The strengths of our study include the large sample which was nationally representative of adults in the US population. Anthropometric data were measured directly by study staff. Blood samples were available for a large proportion of the sample, enabling us to examine the association between dietary pattern scores and CRP. The limitations of this study should also be acknowledged when interpreting the findings. The dietary data were obtained from 24-hour dietary recall interviews and, therefore, subject to considerable measurement error. However, a 24-hour dietary recall is a common tool for measuring nutrient intakes, and alternative self-reported dietary assessment instruments are also subject to this limitation [47, 48]. Additionally, recall bias and omission of food groups may be reduced using this method [49]. The analysis relied on a single day of measurement for dietary data. This may not capture the usual dietary intake of individuals, and dietary supplements were not assessed. To minimize this limitation, we could have additionally utilized NHANES data from the second dietary recall in our analyses. However, restricting our participants to those with two measurements for dietary data would have significantly reduced the sample size. Confounding factors were also limited by self-report assessment, e.g. physical activity. More detailed assessment using more objective measures such as accelerometers may further strengthen findings. The measurement of CRP occurred via latex-enhanced nephelometry for the 1999–2010 survey cycles, a near infrared particle immunoassay rate method for the 2015–2016 survey cycle, and a two-reagent immunoturbidimetric system for the 2017–2018 survey cycle. The contrasting methodologies of measurement across the survey cycles may affect the consistency of the CRP data analysed. However, the distribution of CRP was similar across the survey cycles and the method of CRP measurement (survey cycles) was adjusted for in the analyses. Furthermore, all analyses were cross-sectional and, consequently, causal inference is limited since there was no temporal progression between exposures and outcomes.

We used a relatively small number of food groups compared to other studies applying RRR. This may lead to foods with dissimilar health effects being grouped together and thus the derived dietary patterns may not reflect important differences in dietary intake. Additionally, the utilization of carbohydrate intake as an intermediate response variable may not be ideal as the different sources of the carbohydrates are important and have not been considered. Of note, dietary patterns identified using RRR are based on the population of interest. Therefore, they should not be generalized to other populations with different dietary patterns. It is also worth acknowledging that other dietary components exist that are associated with central obesity and/or systemic inflammation and which will also be correlated with the macronutrients and derived dietary patterns in this study. Consequently, the results of our secondary analyses may be confounded by the intake of other dietary components. It would be of interest to identify and examine the impact of these potential dietary confounders in future studies. Finally, although we minimized confounding bias by adjusting for several potential confounders that were identified a priori using DAGs, confounding by unmeasured factors may remain [50].

Further studies are warranted to address the limitations of our research by applying longitudinal analysis. This approach would allow us to observe changes in dietary patterns that may occur with shifting socioeconomic position over time, and whether such changes result in changes in obesity and inflammation. Future research should also consider using multiple 24-hour recalls instead of just one, in order to present a more comprehensive and accurate picture of participants’ dietary intake.

## Conclusion

In conclusion, four dietary patterns were identified in a population of US adults using RRR, with macronutrients as response variables. We found that economic status had varying associations with the identified dietary patterns. Our findings support the hypothesis that economic status is associated with the relative contribution of macronutrient intakes in the diet, and this in turn may be an important predictor of metabolic health outcomes. Future studies should continue to derive dietary patterns using RRR in different populations and with alternative response variables. Furthermore, RRR should be applied in prospective longitudinal studies to examine the associations between diet and different disease outcomes.

## Electronic supplementary material

Below is the link to the electronic supplementary material.

Supplementary Material 1

## Data Availability

The data used in this paper can be publicly accessed from https://www.cdc.gov/nchs/nhanes/index.htm.

## Abbreviations

CRP: C-reactive protein
DAG: Directed acyclic graph
IQR: Interquartile range
MET: Metabolic equivalent of task
NHANES: National Health and Nutrition Examination Survey
RRR: Reduced rank regression
US: United States
USDA: United States Department of Agriculture
