# Supplementary material for: Dietary patterns derived by reduced rank regression, macronutrients as response variables, and variation by economic status: NHANES 1999–2018
Source: Eur J Nutr. 2024 Sep 17;63(8):3207–21. doi: 10.1007/s00394-024-03501-z (PMC11519099; doi:10.1007/s00394-024-03501-z)
Supplement: Supplementary file 1 — Supplementary Material 1 [file 394_2024_3501_MOESM1_ESM.docx]

**Supplementary Information**

**Dietary patterns derived by reduced rank regression, macronutrients as response variables, and variation by economic status: NHANES 1999-2018**

Samuel C Coxall, Frances EM Albers, Sherly X Li, Zumin Shi, Allison M Hodge, Brigid M Lynch, Yohannes Adama Melaku

**Journal:** European Journal of Nutrition

**Corresponding author:** Brigid M Lynch^1,2,3^ Brigid.Lynch@cancervic.org.au

1 Cancer Epidemiology Division, Cancer Council Victoria, Melbourne, Australia

2 Centre for Epidemiology and Biostatistics, Melbourne School of Population and Global Health, University of Melbourne, Melbourne, Australia

3 Physical Activity Laboratory, Baker Heart and Diabetes Institute, Melbourne, Australia

# **Supplementary Figures**

**
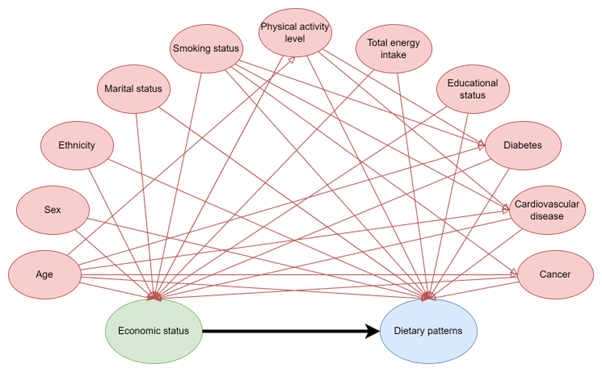
**

**Supplementary Fig. 1** Directed acyclic graph of the association between economic status and dietary patterns


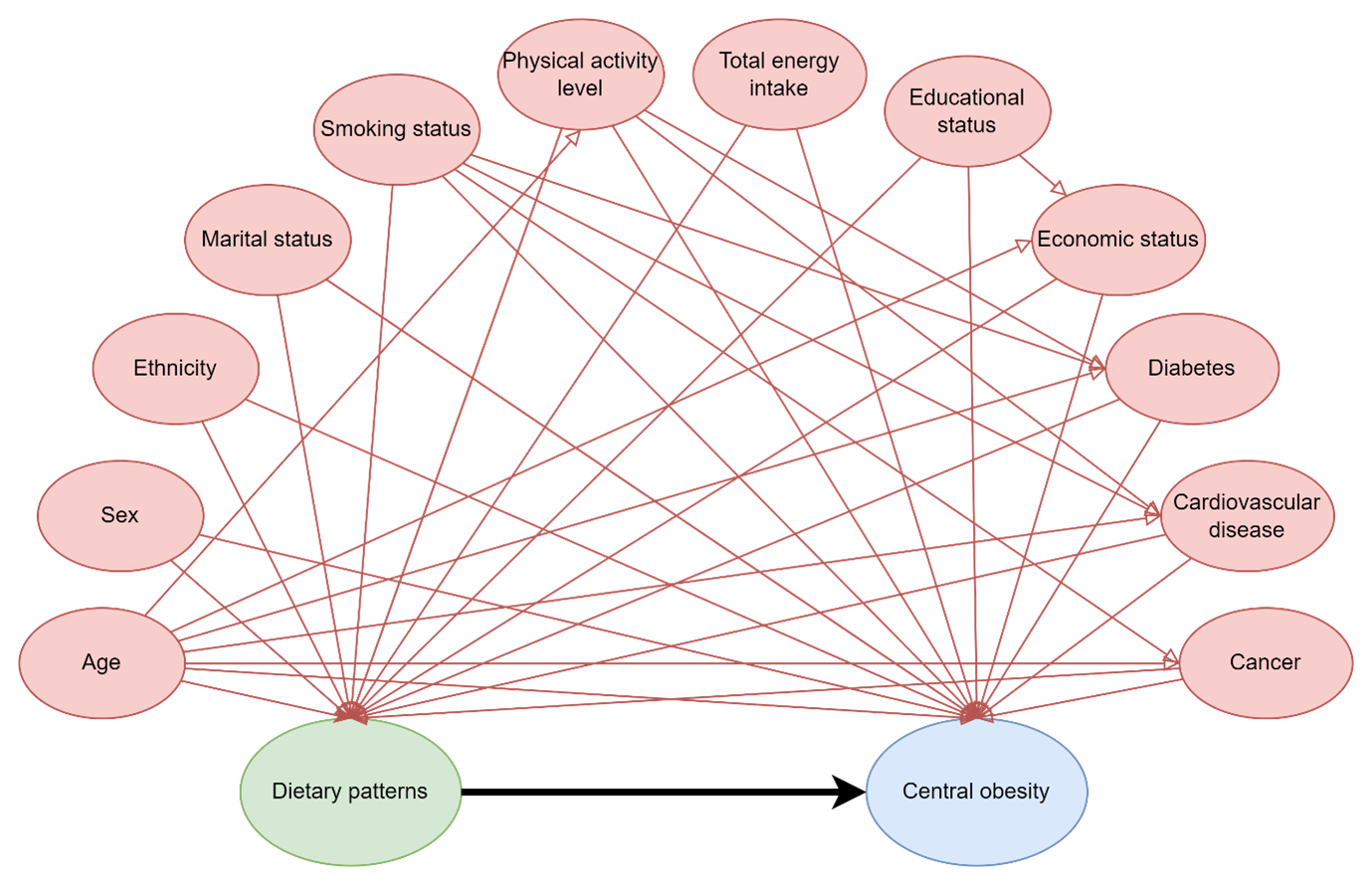


**Supplementary Fig. 2** Directed acyclic graph of the association between dietary patterns and central obesity


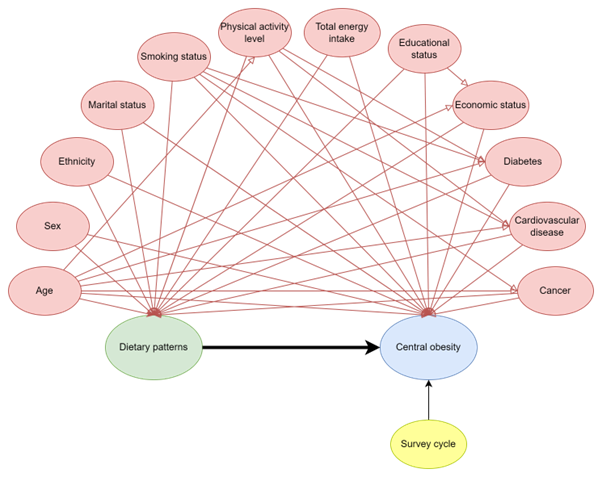


**Supplementary Fig. 3** Directed acyclic graph of the association between dietary patterns and systemic inflammation

# **Supplementary Tables**

| **Supplementary table 1** Characteristics of study participants across quintiles of the high fat, low carbohydrate pattern (n = 39,757) | | | | | |
| --- | --- | --- | --- | --- | --- |
|  | Q1  (n = 7,952) | Q2  (n = 7,951) | Q3  (n = 7,952) | Q4  (n = 7,951) | Q5  (n = 7,951) |
| Age (years), median (IQR) | 43.0 (31.0, 56.0) | 47.0 (33.0, 61.0) | 48.0 (34.0, 62.0) | 48.0 (34.0, 60.0) | 46.0 (33.0, 58.0) |
| Sex, % |  |  |  |  |  |
| Male | 53.8 | 41.2 | 41.4 | 47.1 | 60.1 |
| Female | 46.2 | 58.8 | 58.6 | 52.9 | 39.9 |
| Marital status, % |  |  |  |  |  |
| Married or living with   a partner | 61.3 | 63.3 | 62.6 | 63.2 | 66.0 |
| Separated/divorced | 13.1 | 13.1 | 13.1 | 13.2 | 12.4 |
| Widowed | 4.6 | 6.4 | 7.2 | 6.2 | 4.0 |
| Never married | 21.1 | 17.2 | 17.2 | 17.4 | 17.6 |
| Educational status, % |  |  |  |  |  |
| Less than high school | 20.1 | 16.8 | 16.0 | 14.3 | 12.5 |
| High school diploma  (including GED) | 24.9 | 23.8 | 24.4 | 24.6 | 22.8 |
| More than high school | 55.0 | 59.4 | 59.6 | 61.1 | 64.7 |
| Ethnicity, % |  |  |  |  |  |
| Mexican American | 11.2 | 8.2 | 7.4 | 6.5 | 6.5 |
| Non-Hispanic White | 65.1 | 67.9 | 69.4 | 72.0 | 74.4 |
| Non-Hispanic Black | 9.7 | 10.8 | 10.9 | 10.7 | 10.3 |
| Other Hispanic | 6.1 | 5.8 | 5.2 | 4.9 | 3.8 |
| Other race – including  Multi-Racial | 8.0 | 7.4 | 7.2 | 6.0 | 5.1 |
| Economic status, % |  |  |  |  |  |
| Low | 17.7 | 15.2 | 14.0 | 12.8 | 10.8 |
| Medium | 50.3 | 50.3 | 51.4 | 47.7 | 45.5 |
| High | 32.1 | 34.5 | 34.7 | 39.5 | 43.7 |
| Smoking status, % |  |  |  |  |  |
| Non-smoker | 53.5 | 55.7 | 54.6 | 53.4 | 50.4 |
| Ex-smoker | 21.1 | 23.5 | 24.9 | 26.9 | 28.2 |
| Current smoker | 25.3 | 20.9 | 20.6 | 19.8 | 21.4 |
| Physical activity level, % |  |  |  |  |  |
| Low | 34.3 | 37.8 | 39.0 | 36.6 | 32.9 |
| Moderate | 12.8 | 12.6 | 12.9 | 12.1 | 12.4 |
| High | 52.9 | 49.6 | 48.1 | 51.2 | 54.7 |
| Diabetes, % |  |  |  |  |  |
| Yes | 9.0 | 11.0 | 13.3 | 13.7 | 13.6 |
| No | 91.0 | 89.0 | 86.7 | 86.3 | 86.4 |
| Cardiovascular disease, % |  |  |  |  |  |
| Yes | 8.1 | 9.6 | 9.2 | 8.2 | 7.6 |
| No | 91.9 | 90.4 | 90.8 | 91.8 | 92.4 |
| Cancer, % |  |  |  |  |  |
| Yes | 7.5 | 10.1 | 11.4 | 10.4 | 8.7 |
| No | 92.5 | 89.9 | 88.6 | 89.6 | 91.3 |
| BMI (kg/m2), mean (SD) | 28.2 (6.8) | 28.1 (6.5) | 28.6 (6.6) | 28.9 (6.6) | 29.6 (6.5) |
| Total energy intake   (kJ/day), mean (SD) | 9487.0 (3944.8) | 8011.2 (3437.6) | 7958.1 (3360.9) | 8613.9 (3328.7) | 10905.2 (3656.6) |
| Percent of energy from  protein, mean (SD) | 12.8 (4.2) | 14.7 (4.3) | 15.8 (4.7) | 16.8 (5.0) | 17.8 (5.2) |
| Protein (gm/day), mean  (SD) | 71.9 (36.6) | 68.8 (32.4) | 73.1 (32.5) | 83.7 (34.3) | 112.9 (43.0) |
| Percent of energy from  carbohydrates, mean  (SD) | 62.0 (9.1) | 54.5 (6.7) | 48.9 (6.1) | 43.4 (6.0) | 36.2 (7.0) |
| Carbohydrates (gm/day),   mean (SD) | 344.2 (136.4) | 257.0 (105.9) | 232.5 (103.0) | 227.6 (102.1) | 241.1 (103.7) |
| Percent of energy from  unsaturated fat, mean  (SD) | 13.7 (4.2) | 17.0 (4.0) | 19.6 (4.2) | 22.2 (4.6) | 25.8 (5.6) |
| Unsaturated fat (gm/day),   mean (SD) | 35.6 (20.3) | 36.8 (19.5) | 41.4 (19.7) | 50.0 (20.0) | 73.9 (27.4) |
| Percent of energy from  saturated fat, mean (SD) | 9.9 (3.3) | 12.3 (3.3) | 14.0 (3.3) | 15.8 (3.4) | 18.4 (4.0) |
| Saturated fat (gm/day),   mean (SD) | 25.7 (15.1) | 26.7 (14.6) | 29.5 (14.2) | 35.7 (14.9) | 52.6 (19.9) |
| Waist circumference (cm),   mean (SD) | 96.9 (16.7) | 96.5 (16.1) | 97.8 (16.3) | 99.0 (16.0) | 101.3 (16.0) |
| CRP in the 1999-2010   survey cycles (mg/L),   median (IQR)**^a^** | 1.4 (0.6, 3.1) | 1.6 (0.7, 3.4) | 1.6 (0.7, 3.4) | 1.6 (0.7, 3.3) | 1.6 (0.7, 3.4) |
| CRP in the 2015-2016  survey cycle (mg/L),   median (IQR)**^a^** | 1.6 (0.6, 3.5) | 1.5 (0.6, 3.4) | 1.5 (0.6, 3.3) | 1.8 (0.7, 3.8) | 1.5 (0.6, 3.0) |
| CRP in the 2016-2017  survey cycle (mg/L),   median (IQR)**^a^** | 1.6 (0.9, 3.5) | 1.4 (0.7, 3.2) | 1.9 (0.9, 4.2) | 1.9 (0.9, 3.3) | 1.5 (0.8, 3.0) |
| Data are presented as mean (SD) for normally distributed continuous measures, median (IQR) for non-normally distributed continuous measures, and % for categorical measures.  **^a^** CRP levels are displayed as separate for specific survey cycles as alternative methods for CRP measurement were used.  IQR, interquartile range; GED, General Educational Development; BMI, body mass index; SD, standard deviation; CRP, C-reactive protein. | | | | | |

| **Supplementary table 2** Characteristics of study participants across quintiles of the high protein pattern (n = 39,757) | | | | | |
| --- | --- | --- | --- | --- | --- |
|  | Q1  (n = 7,952) | Q2  (n = 7,951) | Q3  (n = 7,952) | Q4  (n = 7,951) | Q5  (n = 7,951) |
| Age (years), median (IQR) | 45.0 (32.0, 57.0) | 47.0 (33.0, 61.0) | 47.0 (34.0, 61.0) | 47.0 (34.0, 60.0) | 45.0 (33.0, 58.0) |
| Sex, % |  |  |  |  |  |
| Male | 53.6 | 44.4 | 43.4 | 45.3 | 58.3 |
| Female | 46.4 | 55.6 | 56.6 | 54.7 | 41.7 |
| Marital status, % |  |  |  |  |  |
| Married or living with a   partner | 62.6 | 62.1 | 63.6 | 64.1 | 64.5 |
| Separated/divorced | 14.1 | 13.5 | 12.3 | 12.7 | 12.1 |
| Widowed | 4.9 | 6.1 | 6.4 | 6.4 | 4.4 |
| Never married | 18.4 | 18.2 | 17.7 | 16.8 | 19.0 |
| Educational status, % |  |  |  |  |  |
| Less than high school | 14.0 | 15.2 | 17.3 | 16.3 | 16.4 |
| High school diploma  (including GED) | 27.5 | 25.6 | 23.1 | 23.0 | 20.7 |
| More than high school | 58.5 | 59.2 | 59.7 | 60.7 | 62.9 |
| Ethnicity, % |  |  |  |  |  |
| Mexican American | 6.1 | 6.7 | 8.1 | 8.9 | 9.7 |
| Non-Hispanic White | 74.7 | 72.0 | 69.3 | 68.7 | 64.5 |
| Non-Hispanic Black | 11.3 | 10.9 | 10.4 | 9.6 | 10.0 |
| Other Hispanic | 3.5 | 4.6 | 5.4 | 5.4 | 6.9 |
| Other race – including  Multi-Racial | 4.5 | 5.9 | 6.8 | 7.3 | 9.0 |
| Economic status, % |  |  |  |  |  |
| Low | 13.7 | 14.0 | 14.7 | 13.8 | 13.5 |
| Medium | 50.0 | 50.4 | 49.3 | 48.4 | 46.2 |
| High | 36.2 | 35.5 | 35.9 | 37.9 | 40.3 |
| Smoking status, % |  |  |  |  |  |
| Non-smoker | 49.3 | 51.7 | 54.9 | 55.3 | 56.3 |
| Ex-smoker | 25.4 | 25.0 | 24.0 | 24.7 | 26.3 |
| Current smoker | 25.4 | 23.4 | 21.1 | 19.9 | 17.4 |
| Physical activity level, % |  |  |  |  |  |
| Low | 35.4 | 38.0 | 38.3 | 36.0 | 32.5 |
| Moderate | 12.2 | 12.2 | 13.5 | 12.4 | 12.5 |
| High | 52.4 | 49.7 | 48.2 | 51.6 | 55.0 |
| Diabetes, % |  |  |  |  |  |
| Yes | 10.0 | 11.8 | 13.1 | 14.0 | 12.3 |
| No | 90.1 | 88.2 | 86.9 | 86.0 | 87.7 |
| Cardiovascular disease, % |  |  |  |  |  |
| Yes | 7.8 | 8.9 | 9.4 | 8.8 | 7.7 |
| No | 92.3 | 91.1 | 90.7 | 91.2 | 92.4 |
| Cancer, % |  |  |  |  |  |
| Yes | 9.5 | 10.3 | 10.5 | 9.3 | 8.6 |
| No | 90.5 | 89.7 | 89.5 | 90.7 | 91.4 |
| BMI (kg/m2), mean (SD) | 28.8 (6.6) | 28.7 (6.7) | 28.6 (6.6) | 28.7 (6.7) | 28.7 (6.6) |
| Total energy intake   (kJ/day), mean (SD) | 11482.7 (3723.5) | 8576.5 (3262.5) | 8020.6 (3316.8) | 7916.8 (3302.2) | 9024.3 (3759.4) |
| Percent of energy from  protein, mean (SD) | 11.3 (2.5) | 13.0 (2.8) | 14.9 (2.7) | 17.5 (2.9) | 22.3 (5.2) |
| Protein (gm/day), mean  (SD) | 78.8 (33.4) | 68.2 (32.5) | 71.9 (33.2) | 81.3 (34.1) | 116.3 (47.2) |
| Percent of energy from  carbohydrates, mean  (SD) | 48.9 (10.2) | 50.1 (11.1) | 49.9 (11.5) | 48.6 (11.6) | 44.7 (11.7) |
| Carbohydrates (gm/day),   mean (SD) | 334.5 (128.0) | 253.1 (103.2) | 234.8 (102.5) | 226.2 (101.5) | 239.6 (114.2) |
| Percent of energy from  unsaturated fat, mean  (SD) | 23.4 (6.3) | 20.9 (6.0) | 19.2 (5.8) | 18.3 (5.7) | 17.4 (5.5) |
| Unsaturated fat (gm/day),   mean (SD) | 70.5 (28.2) | 47.2 (21.6) | 41.1 (21.2) | 39.0 (21.8) | 42.3 (23.3) |
| Percent of energy from  saturated fat, mean (SD) | 15.6 (4.4) | 14.7 (4.4) | 14.0 (4.6) | 13.5 (4.5) | 13.1 (4.5) |
| Saturated fat (gm/day),   mean (SD) | 47.5 (20.1) | 33.5 (16.7) | 30.2 (16.7) | 28.9 (16.6) | 32.0 (18.5) |
| Waist circumference (cm),   mean (SD) | 99.1 (16.2) | 98.6 (16.1) | 97.9 (16.2) | 98.1 (16.5) | 98.3 (16.5) |
| CRP in the 1999-2010   survey cycles (mg/L),   median (IQR)**^a^** | 1.6 (0.7, 3.3) | 1.6 (0.7, 3.4) | 1.6 (0.7, 3.4) | 1.5 (0.6, 3.4) | 1.5 (0.6, 3.2) |
| CRP in the 2015-2016  survey cycle (mg/L),   median (IQR)**^a^** | 1.7 (0.7, 3.7) | 1.6 (0.6, 3.7) | 1.7 (0.8, 3.5) | 1.6 (0.6, 3.3) | 1.4 (0.6, 2.9) |
| CRP in the 2016-2017  survey cycle (mg/L),   median (IQR)**^a^** | 1.7 (0.8, 3.6) | 1.7 (0.8, 3.9) | 1.9 (0.9, 3.6) | 1.7 (0.8, 3.0) | 1.4 (0.8, 2.9) |
| Data are presented as mean (SD) for normally distributed continuous measures, median (IQR) for non-normally distributed continuous measures, and % for categorical measures.  **^a^** CRP levels are displayed as separate for specific survey cycles as alternative methods for CRP measurement were used.  IQR, interquartile range; GED, General Educational Development; BMI, body mass index; SD, standard deviation; CRP, C-reactive protein. | | | | | |

| **Supplementary table 3** Characteristics of study participants across quintiles of the high saturated fat pattern (n = 39,757) | | | | | |
| --- | --- | --- | --- | --- | --- |
|  | Q1  (n = 7,952) | Q2  (n = 7,951) | Q3  (n = 7,952) | Q4  (n = 7,951) | Q5  (n = 7,951) |
| Age (years), median (IQR) | 47.0 (34.0, 58.0) | 47.0 (34.0, 60.0) | 48.0 (35.0, 62.0) | 47.0 (33.0, 61.0) | 42.0 (31.0, 56.0) |
| Sex, % |  |  |  |  |  |
| Male | 57.2 | 44.6 | 42.8 | 42.2 | 57.0 |
| Female | 42.8 | 55.4 | 57.2 | 57.8 | 43.0 |
| Marital status, % |  |  |  |  |  |
| Married or living with a   partner | 65.1 | 64.0 | 65.0 | 61.6 | 61.4 |
| Separated/divorced | 11.8 | 13.6 | 12.3 | 13.0 | 14.0 |
| Widowed | 4.3 | 5.8 | 6.6 | 7.2 | 4.4 |
| Never married | 18.8 | 16.5 | 16.1 | 18.3 | 20.2 |
| Educational status, % |  |  |  |  |  |
| Less than high school | 12.2 | 15.0 | 17.7 | 17.9 | 16.4 |
| High school diploma  (including GED) | 20.7 | 23.5 | 23.8 | 25.1 | 27.0 |
| More than high school | 67.1 | 61.5 | 58.5 | 57.0 | 56.6 |
| Ethnicity, % |  |  |  |  |  |
| Mexican American | 7.8 | 8.1 | 8.4 | 7.7 | 7.4 |
| Non-Hispanic White | 65.8 | 65.9 | 68.6 | 71.8 | 76.8 |
| Non-Hispanic Black | 11.8 | 11.4 | 11.5 | 9.7 | 8.3 |
| Other Hispanic | 5.0 | 5.4 | 5.2 | 5.9 | 4.1 |
| Other race – including  Multi-Racial | 9.6 | 9.3 | 6.3 | 4.9 | 3.5 |
| Economic status, % |  |  |  |  |  |
| Low | 11.9 | 14.2 | 14.0 | 15.2 | 14.4 |
| Medium | 45.3 | 47.6 | 49.0 | 50.2 | 52.3 |
| High | 42.8 | 38.2 | 37.0 | 34.6 | 33.3 |
| Smoking status, % |  |  |  |  |  |
| Non-smoker | 53.2 | 54.8 | 54.9 | 54.1 | 50.4 |
| Ex-smoker | 27.8 | 25.7 | 24.8 | 23.9 | 23.3 |
| Current smoker | 19.0 | 19.5 | 20.3 | 22.0 | 26.4 |
| Physical activity level, % |  |  |  |  |  |
| Low | 31.0 | 37.3 | 37.3 | 39.0 | 36.0 |
| Moderate | 11.3 | 12.7 | 13.0 | 13.6 | 12.4 |
| High | 57.7 | 50.1 | 49.8 | 47.5 | 51.7 |
| Diabetes, % |  |  |  |  |  |
| Yes | 11.3 | 13.3 | 13.5 | 12.4 | 10.7 |
| No | 88.7 | 86.7 | 86.5 | 87.6 | 89.3 |
| Cardiovascular disease, % |  |  |  |  |  |
| Yes | 6.8 | 8.9 | 9.9 | 9.5 | 7.6 |
| No | 93.2 | 91.1 | 90.2 | 90.5 | 92.4 |
| Cancer, % |  |  |  |  |  |
| Yes | 8.5 | 10.6 | 10.2 | 10.6 | 8.6 |
| No | 91.6 | 89.4 | 89.8 | 89.4 | 91.4 |
| BMI (kg/m2), mean (SD) | 28.4 (6.4) | 28.5 (6.6) | 28.8 (6.9) | 28.8 (6.7) | 29.0 (6.6) |
| Total energy intake   (kJ/day), mean (SD) | 10244.5 (3770.9) | 8223.0 (3267.0) | 7745.4 (3372.3) | 8138.6 (3325.6) | 10603.6 (3783.8) |
| Percent of energy from  protein, mean (SD) | 15.7 (5.5) | 15.8 (5.5) | 15.7 (5.3) | 15.7 (5.0) | 15.6 (4.2) |
| Protein (gm/day), mean  (SD) | 93.7 (42.9) | 76.0 (36.5) | 71.1 (35.6) | 74.7 (34.9) | 97.5 (41.0) |
| Percent of energy from  carbohydrates, mean  (SD) | 44.3 (10.7) | 49.2 (11.7) | 51.0 (12.0) | 50.5 (11.1) | 47.5 (10.2) |
| Carbohydrates (gm/day),   mean (SD) | 271.0 (118.9) | 239.8 (107.4) | 233.2 (109.7) | 243.6 (109.0) | 301.1 (125.9) |
| Percent of energy from  unsaturated fat, mean  (SD) | 24.2 (7.3) | 20.0 (6.3) | 18.6 (5.8) | 18.2 (5.2) | 18.6 (4.6) |
| Unsaturated fat (gm/day),   mean (SD) | 65.0 (30.2) | 43.9 (22.6) | 38.9 (22.3) | 40.0 (21.3) | 52.9 (24.2) |
| Percent of energy from  saturated fat, mean (SD) | 12.2 (3.6) | 12.5 (4.0) | 13.4 (4.2) | 15.1 (4.1) | 17.7 (4.3) |
| Saturated fat (gm/day),   mean (SD) | 33.4 (16.7) | 27.9 (15.5) | 28.1 (16.3) | 32.5 (15.9) | 49.4 (20.6) |
| Waist circumference (cm),   mean (SD) | 98.0 (15.9) | 97.7 (16.3) | 98.4 (16.7) | 98.2 (16.3) | 99.7 (16.2) |
| CRP in the 1999-2010   survey cycles (mg/L),   median (IQR)**^a^** | 1.2 (0.5, 2.8) | 1.5 (0.6, 3.2) | 1.7 (0.7, 3.4) | 1.7 (0.7, 3.6) | 1.7 (0.7, 3.5) |
| CRP in the 2015-2016  survey cycle (mg/L),   median (IQR)**^a^** | 1.4 (0.6, 3.0) | 1.4 (0.6, 3.1) | 1.6 (0.6, 3.5) | 1.9 (0.8, 4.0) | 1.8 (0.8, 3.7) |
| CRP in the 2016-2017  survey cycle (mg/L),   median (IQR)**^a^** | 1.5 (0.7, 3.1) | 1.6 (0.7, 3.6) | 1.9 (0.9, 3.4) | 1.7 (1.0, 3.7) | 2.0 (0.9, 3.9) |
| Data are presented as mean (SD) for normally distributed continuous measures, median (IQR) for non-normally distributed continuous measures, and % for categorical measures.  **^a^** CRP levels are displayed as separate for specific survey cycles as alternative methods for CRP measurement were used.  IQR, interquartile range; GED, General Educational Development; BMI, body mass index; SD, standard deviation; CRP, C-reactive protein. | | | | | |

| **Supplementary table 4** Characteristics of study participants across quintiles of the Low alcohol pattern (n = 39,757) | | | | | |
| --- | --- | --- | --- | --- | --- |
|  | Q1  (n = 7,952) | Q2  (n = 7,951) | Q3  (n = 7,952) | Q4  (n = 7,951) | Q5  (n = 7,951) |
| Age (years), median (IQR) | 44.0 (33.0, 56.0) | 45.0 (32.0, 60.0) | 47.0 (33.0, 61.0) | 48.0 (34.0, 62.0) | 48.0 (35.0, 59.0) |
| Sex, % |  |  |  |  |  |
| Male | 62.3 | 39.8 | 39.9 | 45.5 | 55.9 |
| Female | 37.7 | 60.2 | 60.2 | 54.6 | 44.1 |
| Marital status, % |  |  |  |  |  |
| Married or living with a   partner | 63.1 | 59.8 | 61.9 | 64.9 | 66.9 |
| Separated/divorced | 14.3 | 14.1 | 13.4 | 11.8 | 11.1 |
| Widowed | 3.5 | 6.4 | 7.2 | 6.6 | 4.7 |
| Never married | 19.1 | 19.7 | 17.5 | 16.6 | 17.2 |
| Educational status, % |  |  |  |  |  |
| Less than high school | 12.0 | 19.4 | 18.3 | 16.6 | 13.3 |
| High school diploma  (including GED) | 22.9 | 27.1 | 26.2 | 23.5 | 20.8 |
| More than high school | 65.1 | 53.5 | 55.5 | 60.0 | 65.9 |
| Ethnicity, % |  |  |  |  |  |
| Mexican American | 5.9 | 7.2 | 7.9 | 9.5 | 9.0 |
| Non-Hispanic White | 76.4 | 71.9 | 68.6 | 66.2 | 65.9 |
| Non-Hispanic Black | 9.3 | 11.1 | 11.5 | 11.4 | 9.3 |
| Other Hispanic | 4.1 | 5.1 | 5.4 | 5.7 | 5.4 |
| Other race – including  Multi-Racial | 4.3 | 4.7 | 6.7 | 7.3 | 10.4 |
| Economic status, % |  |  |  |  |  |
| Low | 10.4 | 17.6 | 15.7 | 13.9 | 12.6 |
| Medium | 43.3 | 51.4 | 52.2 | 51.9 | 46.6 |
| High | 46.3 | 31.1 | 32.1 | 34.2 | 40.8 |
| Smoking status, % |  |  |  |  |  |
| Non-smoker | 41.1 | 51.9 | 56.4 | 59.0 | 59.8 |
| Ex-smoker | 28.5 | 22.5 | 22.2 | 24.9 | 26.7 |
| Current smoker | 30.4 | 25.6 | 21.3 | 16.1 | 13.4 |
| Physical activity level, % |  |  |  |  |  |
| Low | 29.7 | 41.6 | 40.8 | 38.0 | 31.1 |
| Moderate | 13.1 | 12.3 | 12.6 | 13.3 | 11.5 |
| High | 57.3 | 46.2 | 46.6 | 48.7 | 57.3 |
| Diabetes, % |  |  |  |  |  |
| Yes | 7.8 | 13.1 | 14.5 | 13.9 | 12.4 |
| No | 92.2 | 87.0 | 85.5 | 86.2 | 87.6 |
| Cardiovascular disease, % |  |  |  |  |  |
| Yes | 6.1 | 9.5 | 9.6 | 9.6 | 7.9 |
| No | 93.9 | 90.5 | 90.4 | 90.4 | 92.1 |
| Cancer, % |  |  |  |  |  |
| Yes | 8.6 | 9.7 | 9.9 | 10.6 | 9.5 |
| No | 91.4 | 90.3 | 90.1 | 89.4 | 90.5 |
| BMI (kg/m2), mean (SD) | 27.7 (5.6) | 29.2 (7.0) | 29.3 (7.1) | 29.1 (6.9) | 28.5 (6.4) |
| Total energy intake   (kJ/day), mean (SD) | 10290.7 (3804.2) | 7744.2 (3509.2) | 7789.6 (3201.6) | 8699.4 (3238.7) | 10495.7 (3756.8) |
| Percent of energy from  protein, mean (SD) | 14.6 (4.3) | 15.3 (4.7) | 15.8 (5.2) | 16.1 (5.3) | 16.8 (5.6) |
| Protein (gm/day), mean  (SD) | 88.5 (39.7) | 69.8 (36.6) | 71.6 (33.4) | 81.1 (34.4) | 103.0 (44.5) |
| Percent of energy from  carbohydrates, mean  (SD) | 41.0 (9.6) | 48.2 (10.6) | 51.1 (10.9) | 51.7 (10.8) | 51.1 (11.2) |
| Carbohydrates (gm/day),   mean (SD) | 252.1 (110.6) | 220.3 (106.4) | 235.4 (103.4) | 267.1 (109.6) | 318.6 (131.3) |
| Percent of energy from  unsaturated fat, mean  (SD) | 18.2 (5.6) | 20.0 (5.5) | 19.7 (5.9) | 20.1 (6.3) | 21.7 (7.3) |
| Unsaturated fat (gm/day),   mean (SD) | 50.3 (25.4) | 41.7 (23.0) | 41.3 (22.1) | 47.0 (23.7) | 61.0 (30.9) |
| Percent of energy from  saturated fat, mean (SD) | 13.6 (4.7) | 17.0 (4.7) | 14.6 (4.3) | 13.6 (3.9) | 12.5 (3.6) |
| Saturated fat (gm/day),   mean (SD) | 37.7 (20.0) | 35.6 (20.2) | 31.3 (17.9) | 32.4 (17.5) | 35.7 (18.6) |
| Waist circumference (cm),   mean (SD) | 97.2 (14.5) | 99.1 (16.7) | 99.1 (17.1) | 99.0 (16.9) | 98.0 (16.4) |
| CRP in the 1999-2010   survey cycles (mg/L),   median (IQR)**^a^** | 1.3 (0.6, 2.8) | 1.8 (0.7, 3.9) | 1.8 (0.7, 3.5) | 1.7 (0.7, 3.6) | 1.3 (0.6, 2.9) |
| CRP in the 2015-2016  survey cycle (mg/L),   median (IQR)**^a^** | 1.4 (0.6, 2.9) | 2.2 (0.9, 4.4) | 1.7 (0.7, 3.4) | 1.6 (0.6, 3.3) | 1.3 (0.6, 3.0) |
| CRP in the 2016-2017  survey cycle (mg/L),   median (IQR)**^a^** | 1.5 (0.8, 3.5) | 1.7 (0.9, 3.5) | 2.0 (1.0, 3.6) | 1.9 (1.0, 4.0) | 1.3 (0.7, 3.0) |
| Data are presented as mean (SD) for normally distributed continuous measures, median (IQR) for non-normally distributed continuous measures, and % for categorical measures.  **^a^** CRP levels are displayed as separate for specific survey cycles as alternative methods for CRP measurement were used.  IQR, interquartile range; GED, General Educational Development; BMI, body mass index; SD, standard deviation; CRP, C-reactive protein. | | | | | |

| **Supplementary table 5** Coefficients (β) and 95% confidence intervals of waist circumference (cm) across quintiles of dietary pattern scores after adjusting for confounders, stratified by sex (n = 39,757) | | | | | | |
| --- | --- | --- | --- | --- | --- | --- |
| Dietary patterns | β (95% CI) | | | | |  |
|  | Q1 | Q2 | Q3 | Q4 | Q5 | *P for trend* |
| **Male (n =** **19,854)** |  |  |  |  |  |  |
| High fat, low   carbohydrate pattern | Ref. | -0.02 (-1.02, 0.98) | 0.76 (-0.06, 1.58) | 1.66 (0.73, 2.59) | 3.38 (2.48, 4.28) | <0.001 |
| High protein pattern | Ref. | 0.46 (-0.35, 1.27) | 0.05 (-0.81, 0.91) | -0.31 (-1.16, 0.55) | -0.15 (-1.09, 0.79) | 0.379 |
| High saturated fat pattern | Ref. | 0.33 (-0.57, 1.23) | 0.47 (-0.40, 1.34) | 0.72 (-0.29, 1.72) | 1.63 (0.78, 2.48) | <0.001 |
| Low alcohol pattern | Ref. | 2.26 (1.37, 3.15) | 2.06 (1.10, 3.01) | 1.24 (0.37, 2.10) | 0.27 (-0.60, 1.14) | 0.804 |
| **Female (n = 19,903)** |  |  |  |  |  |  |
| High fat, low   carbohydrate pattern | Ref. | -0.49 (-1.44, 0.46) | 0.56 (-0.45, 1.57) | 1.20 (0.25, 2.15) | 2.63 (1.32, 3.94) | <0.001 |
| High protein pattern | Ref. | -0.09 (-1.11, 0.92) | -0.72 (-1.81, 0.36) | -0.31 (-1.43, 0.80) | -0.58 (-1.55, 0.39) | 0.214 |
| High saturated fat pattern | Ref. | 0.11 (-1.00, 1.21) | 1.10 (-0.06, 2.26) | 0.71 (-0.32-1.74) | 1.83 (0.68, 2.98) | 0.001 |
| Low alcohol pattern | Ref. | 3.13 (2.22, 4.04) | 2.93 (2.00, 3.85) | 2.32 (1.38, 3.27) | 0.47 (-0.61, 1.55) | 0.837 |
| Adjusted for age, educational status, marital status, ethnicity, economic status, smoking status, physical activity level, total energy intake, diabetes, cardiovascular disease, and cancer.  *P for trend* was calculated by including the quintiles of factor scores as continuous variables. | | | | | | |
